# Genetic Interaction between *Mfrp* and *Adipor1* Mutations Affect Retinal Disease Phenotypes

**DOI:** 10.3390/ijms23031615

**Published:** 2022-01-30

**Authors:** Navdeep Gogna, Sonia Weatherly, Fuxin Zhao, Gayle B. Collin, Jai Pinkney, Lisa Stone, Jürgen K. Naggert, Gregory W. Carter, Patsy M. Nishina

**Affiliations:** 1 The Jackson Laboratory, 600 Main Street, Bar Harbor, ME 04609, USA; navdeep.gogna@jax.org (N.G.); soniawxly@gmail.com (S.W.); zhaofuxing12@163.com (F.Z.); gayle.collin@jax.org (G.B.C.); jai.pinkney@gmail.com (J.P.); lisa.stone@jax.org (L.S.); juergen.naggert@jax.org (J.K.N.); gregory.carter@jax.org (G.W.C.); 2 School of Optometry and Ophthalmology and Eye Hospital, Wenzhou Medical University, Wenzhou 325027, China

**Keywords:** *Mfrp*, *Adipor1*, genetic interaction, fundus spots, photoreceptor degeneration, axial length

## Abstract

*Adipor1^tm1Dgen^* and *Mfrp^rd6^* mutant mice share similar eye disease characteristics. Previously, studies established a functional relationship of ADIPOR1 and MFRP proteins in maintaining retinal lipidome homeostasis and visual function. However, the independent and/or interactive contribution of both genes to similar disease phenotypes, including fundus spots, decreased axial length, and photoreceptor degeneration has yet to be examined. We performed a gene-interaction study where homozygous *Adipor1^tm1Dgen^* and *Mfrp^rd6^* mice were bred together and the resulting doubly heterozygous F1 offspring were intercrossed to produce 210 F2 progeny. Four-month-old mice from all nine genotypic combinations obtained in the F2 generation were assessed for white spots by fundus photo documentation, for axial length by caliper measurements, and for photoreceptor degeneration by histology. Two-way factorial ANOVA was performed to study individual as well as gene interaction effects on each phenotype. Here, we report the first observation of reduced axial length in *Adipor1^tmlDgen^* homozygotes. We show that while *Adipor1* and *Mfrp* interact to affect spotting and degeneration, they act independently to control axial length, highlighting the complex functional association between these two genes. Further examination of the molecular basis of this interaction may help in uncovering mechanisms by which these genes perturb ocular homeostasis.

## 1. Introduction

Membrane-type frizzled-related protein (MFRP), a transmembrane protein with two cubilin (CUB), a low-density lipoprotein receptor a (LDLRA), and a cysteine-rich (CRD) [1] domain, is expressed and localized in the retinal pigment epithelium (RPE) and ciliary body epithelium [2,3] of the eye. Disruptions in *MFRP* are known to cause a spectrum of human ocular diseases, including hyperopia [4], nanophthalmos [5,6], posterior microphthalmos [7], retinitis pigmentosa [8], foveoschisis [9,10], and optic disc drusen [11]. Likewise, mouse models with mutations in *Mfrp*, including *rd6* (4 bp deletion) [2], *rdx* (174delG mutation) [12], and a c.498_499insC knock-in [13], show phenotypic similarities to the human diseases such as hyperopia, reduced axial length, retinal degeneration, RPE atrophy, and decreased electrophysiological response. The *Mfrp* mouse models also show the presence of uniformly sized and evenly distributed white spots across the fundus, similar to the white flecks observed in the patients with *MFRP* mutations [14,15] and in the human flecked retinal disorder retinitis punctata albescens [2,16].

In mouse, mutations in another gene, adiponectin receptor 1 (*Adipor1*), which encodes an integral membrane protein that localizes to RPE and photoreceptor (PR) cells [17], show disease characteristics similar to those found in *Mfrp* mouse models [12,13,17,18,19]. Mutations in *Adipor1* also result in fundus spots and PR cell death [17]. While *Mfrp* is known to affect ocular growth [4,20,21], the same has not been studied in *Adipor1* mutants. Mutations in *ADIPOR1* have been associated with retinitis pigmentosa [18,19] and age-related macular degeneration [22]. While studies suggest that both ADIPOR1 and MFRP participate in docosahexaenoic acid (DHA, 22:6) enrichment [23] and lipid homeostasis in RPE and PR cells [24], there is still an incomplete understanding about the function of the individual proteins and their potential functional association and interaction that lead to similar disease phenotypes.

Quantitative genetic interaction/epistasis studies are a useful approach to examine functional relationships between genes and pathways [25,26,27]. Epistatic studies aim to determine the extent to which a particular phenotype in a double gene mutant varies from the phenotype observed in individual, single gene mutants [28]. However, such interaction studies are faced with complex interpretations when gene variants affect multiple phenotypes (pleiotrophy) and biological processes. Epistasis found for one phenotype may not be observed for another phenotype, making the interpretation about genetic interaction between two genes more complicated. As a first step towards unraveling the functional association between *Adipor1* and *Mfrp*, our study aimed to determine whether any of the ocular disease phenotypes were attributable to epistatic effects between mutations in these genes. Here, we performed a phenotype/genotype assessment of disease phenotypes observed in *Adipor1^tm1Dgen^* and *Mfrp^rd6^* mice and their double mutant (*Adipor1^tm1Dgen^* × *Mfrp^rd6^*) F2 intercross progeny. Using statistical approaches, we examined individual genes as well as gene interaction effects on three phenotypes, including fundus spotting, axial length (AL), and PR outer nuclear layer (ONL) thickness. This study underscores the importance of epistatic studies in interpreting complex functional relationships between two genes that share multiple similar phenotypes.

## 2. Results

### 2.1. Phenotypic Similarities between Mfrp^rd6^ and Adipor1^tm1Dgen^ Mice

**Figure 1:**
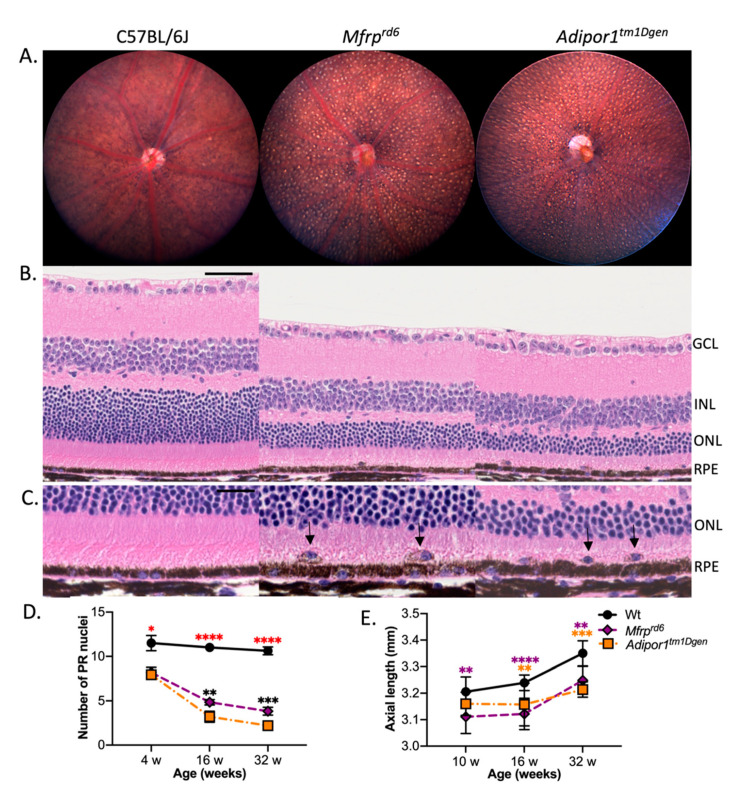
*Mfrp^rd6^* and *Adipor1^tm1Dgen^* mutant mice have similar ocular phenotypes. (**A**) Fundus photographs show evenly distributed discrete white spots throughout the retina of 4-month-old homozygous *Mfrp^rd6^* and *Adipor1^tm1Dgen^* mice, respectively, as compared to age-matched C57BL/6J controls. *n* = 9–12 per strain; both sexes included. (**B**) Retinal sections stained with hematoxylin and eosin (H&E) and visualized by light microscopy reveal significant photoreceptor degeneration by 4-months of age in both mutants compared to age-matched C57BL/6J controls. Scale bar: 50 microns (**C**). Aberrant nucleated cells containing melanin pigment, similar to MOMA2+ pigmented cells described by Hawes et al. [16], are observed in the subretinal space (black arrows) of mutant retinas only. *n* = 10 for each strain. Scale bar: 20 microns. Longitudinal quantification of (**D**) photoreceptor degeneration (#PR nuclei/300 µm length) and (**E**) axial length changes in single homozygotes from (*Adipor^tm1Dgen^* x *Mfrp^rd6^*) F2 progeny: orange, homozygous for *Adipor1^tm1Dgen^* and wt for *Mfrp^rd6^*; purple, wt for *Adipor1^tm1Dgen^* and homozygous for *Mfrp^rd6^*; and black, wt for both *Adipor1^tm1Dgen^* and *Mfrp^rd6^*. Asterisks (*) represent the level of significance (ANOVA) between mutant and control mice; red asterisks indicate that both strains shared the same degree of significance with respect to the control, orange asterisks indicate significance between wt and *Adipor1^tm1Dgen^*, purple asterisks indicate significance between wt and *Mfrp^rd6^*, and black asterisks indicate significance between *Adipor1^tm1Dgen^* and *Mfrp^rd6^*. * *p* < 0.05, ** *p* > 0.01, *** *p* > 0.001, **** *p* > 0.0001. For PR degeneration, *n* = 4–6; for axial length, *n* = 8–15 mice. Both sexes combined.

Both *Mfrp^rd6^* and *Adipor^tm1Dgen^* homozygotes present with small, uniformly sized white spots distributed pan-retinally (4-month-old depicted in Figure 1A). Outer nuclear layer thickness decreases relative to controls as photoreceptors undergo cell death (Figure 1B). Pigmented cells are found ectopically in the subretinal space at the PR and RPE interface (Figure 1C). A longitudinal study of the rate of PR degeneration in homozygous *Adipor^tm1Dgen^* or *Mfrp^rd6^* mutants obtained from a (*Adipor^tm1Dgen^* × *Mfrp^rd6^*) F2 cross indicates that the PR degeneration in homozygous *Adipor1 **^tm1Dgen^*** mutants occurs at a slightly faster rate than in *Mfrp^rd6^* mutant mice of the same age (Figure 1D).

Axial length (AL), a key determinant of the refractive state of the eye, was also examined. A reduction in AL was observed in single homozygotes relative to controls by 10 weeks of age and progressed, such that by 4 months of age it was significantly different for both single mutants when compared to WT controls (Figure 1E). Refractive errors occur when light cannot focus properly on the retina, due to the altered shape of the eye. It can be caused by changes in axial length, or shape of the cornea or lens. Since AL is suggested to be a major contributor of refractive error [29] and we observed a change in AL for both the mutants, we also assessed refractive error. Our measurement of refractive error using an infrared photorefractor also confirmed significant changes (*p*-value < 0.0001) in refraction at 10 weeks of age (Supplementary Figure S1), when the refractive errors of both *Adipor1 **^tm1Dgen^*** and *Mfrp^rd6^* homozygotes shifted towards hyperopia.

### 2.2. Effects of Adipor1^tm1Dgen^ and/or Mfrp^rd6^ Alleles on Fundus Appearance

**Figure 2:**
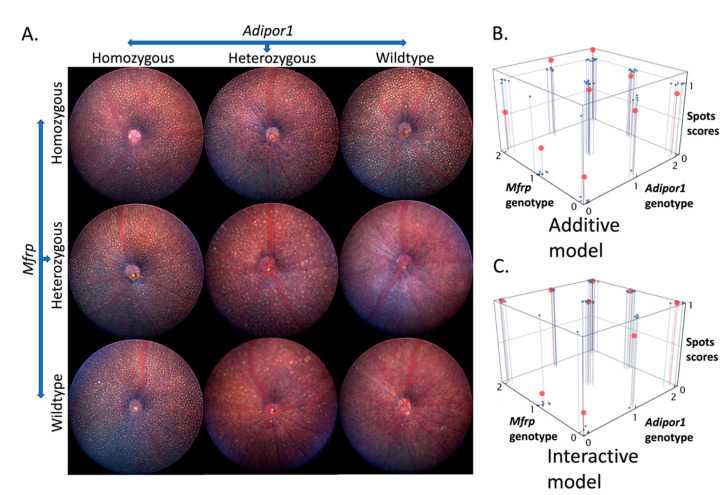
Fundus spots in 4-month-old F2 progeny, obtained from (*Adipor1*^+/−^/*Mfrp^+/rd6^*) F1 intercross. (**A**) Fundus photographs for mutants bearing one of the nine possible combinations of mutations, showing the presence of fundus spots in single and double homozygous mutants as well as double heterozygous mutants. Spots were also observed in heterozygous *Adipor1^+/−^* mice. (**B**) Linear fit model without interactions and (**C**) linear fit model with interactions, showing actual data in blue and model predictions in red. The *Adipor1* and *Mfrp* genotypes along the two axis represent independent variables and the alleles are scored as: wildtype—0, heterozygous—1, and homozygous—2. The scores for fundus spots along the third axis represent response variables and are scored as 0 or 1. Model predictions have a better fit with the actual data in the interactive model as compared to the additive model, confirming an interactive effect. *n* = 5–10 per genotype.

The similarity in *Adipor1**^tm1Dgen^*** and *Mfrp^rd6^* fundus spotting phenotypes raised the possibility that these genes might interact in the same disease process. To test for such an interaction, we intercrossed the two mutants and examined the fundus phenotype at four months of age (Figure 2). In F2 progeny, results of funduscopic examination are shown in Figure 2A with the presence of panretinal white spots in singly homozygous *Adipor1**^tm1Dgen^*** and *Mfrp^rd6^* as well as doubly homozygous (*Adipor1/Mfrp*) mutants. Fundus spots were also seen in heterozygous *Adipor1^+/−^* and double heterozygous *Adipor1^+/−^*/*Mfrp^+/rd6^* mice but not in heterozygous *Mfrp^+/rd6^* mice. To determine if the spotting frequency among the genotypically unique cohorts significantly differed from that of wild type mice, all nine genotypes of F2 mice were evaluated in a masked fashion (central fundus images, estimated to encompass ~1/6th of the entire eye, were obtained and identified by code numbers, randomized, and evaluated without knowledge of the genotype at the time of scoring) on an ordinal scale of 0–1, where less than or equal to five spots was represented as 0 and the presence of more than five uniformly sized spots was represented as 1.

To examine the effect of individual gene mutations and their interaction to affect fundus spots, for the design of the experiment, a full factorial approach with a standard least squares model was used to construct a linear model in JMP statistical analysis software, through the fit model function. The model for spots phenotype had an adjusted R^2^ (goodness-of-fit measure between the model and the dependent variable) value of 0.7056, root mean square error (RMSE; measures how accurately the model predicts the response and has the same unit as the quantity being measured) of 0.2160 and a significant F Ratio (ratio of the variation explained by the model and the unexplained variation) of 19.2792 (prob > F = <0.0001). The effects test confirmed the individual gene effect as well as gene interaction effect on the fundus spots phenotype (Supplementary Table S1). The results were also validated by performing linear regression analysis using the linear model function in R, where the additive and interaction models were generated and compared to find which model of the two was a better fit for the fundus spots data. The comparison between additive and interactive modeling confirmed the interaction model to be a better fit than the purely additive model as shown in Figure 2B,C (adjusted R^2^ value for additive vs. interacting models: 0.5169 vs. 0.7056). An F-ratio test of the F-statistic derived from the two statistical models was significant, indicating superior performance of the interacting model (F-statistic and *p* values: 10.9 and *p* = 1.2 × 10^−6^, respectively). Given that heterozygous *Adipor1^+/−^* mice exhibit spots, statistical analysis also confirmed a significant effect of the heterozygous genotype for *Adipor1* (Supplementary Table S2). However, the spots appear earlier and in greater numbers in the double heterozygous mice than in heterozygous *Adipor1^+/−^* alone (by approximately 10 weeks of age, many spots were seen in double heterozygous mice whereas very few to none were observed in heterozygous *Adipor1^+/−^* mice at the same age. Representative image shown in Supplementary Figure S2), suggesting an epistatic interaction effect between *Adipor1* and *Mfrp* gene variants for the severity of fundus spots. For the *Mfrp^rd6^* allele, no differences were observed between wild type and heterozygous *Mfrp^+/rd6^* mice, whereas homozygous mutants showed fundus spots, confirming the recessive mode of inheritance of the *Mfrp^rd6^* allele for the spotting phenotype.

### 2.3. Effects of Adipor1^tm1Dgen^ and/or Mfrp^rd6^ Alleles on Axial Length (AL)

**Figure 3:**
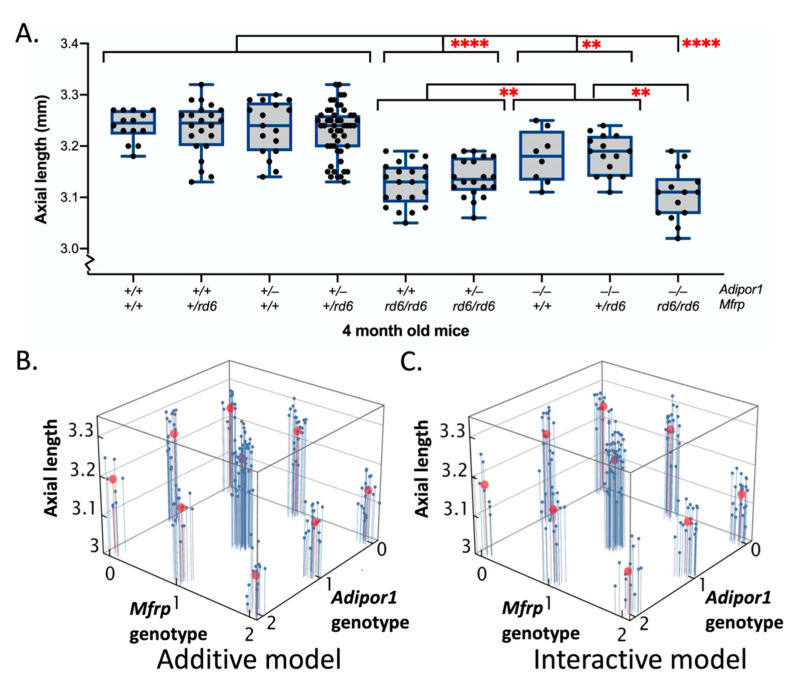
Axial length in 4-month-old F2 progeny obtained from an (*Adipor1*^+/−^/*Mfrp^+/rd6^*) F1 intercross. (**A**) Using two-way factorial ANOVA, followed by Tukey’s HSD post-hoc analysis revealed statistically significant differences in axial length between different genotypic possibilities for the F2 intercross progeny. As no sex-based differences were found, data from both sexes were combined. ** *p* < 0.01; and **** *p* < 0.0001. (**B**) Linear fit model without interactions and (**C**) linear fit model with interactions, showing actual data in blue and model predictions in red. The *Adipor1* and *Mfrp* genotypes along the two axis represent independent variables, where the alleles are scored as: wildtype—0, heterozygous—1, and homozygous—2. The axial length measurements along the third axis represent response variables. The interactive model does not have a better fit than the additive model, confirming no interaction and independent effects. *n* = 14–50 per genotype.

To study the effect of individual gene mutations as well as their interaction on AL changes, the full factorial approach, similar to the one used for fundus spot analysis, was used for the design of the experiment, and a standard least squares approach was used to construct a linear model in JMP software to statistically analyze the ALs of all nine possible genotypes. The interaction model obtained for AL had an adjusted R^2^ value of 0.5160, RMSE value of 0.0461, and a significant F Ratio value of 24.9958 (prob > F = <0.0001). The effects tests confirmed individual gene contributions to the AL (Supplementary Table S3). However, the gene interaction effect was not significant (prob > F = 0.5715). Tukey’s HSD post-hoc analysis revealed significant changes among nine double gene genotypes as shown in Figure 3A. The parameter estimates obtained using statistical analysis (Supplementary Table S4) showed no significant effect of the zygosity in case of either *Adipor1* or *Mfrp* mutant alleles, confirming the recessive nature of inheritance for decreased AL for both genes. The results were also validated by linear modeling using the R function where the gene interaction model was not a better fit than the purely additive model as shown in Figure 3B,C (adjusted R^2^ value for additive vs. interacting models: 0.519 vs. 0.516). An F-ratio test of the F-statistic derived from the two statistical models was not significant, indicating that the interacting model was not a significantly better fit than the additive model and there was no epistasis for the AL phenotype (F-statistic and *p* values: 43.7 and *p* = 0.56, respectively).

### 2.4. Effects of Adipor1^tm1Dgen^ and/or Mfrp^rd6^ Alleles on Photoreceptor (PR) Degeneration

**Figure 4:**
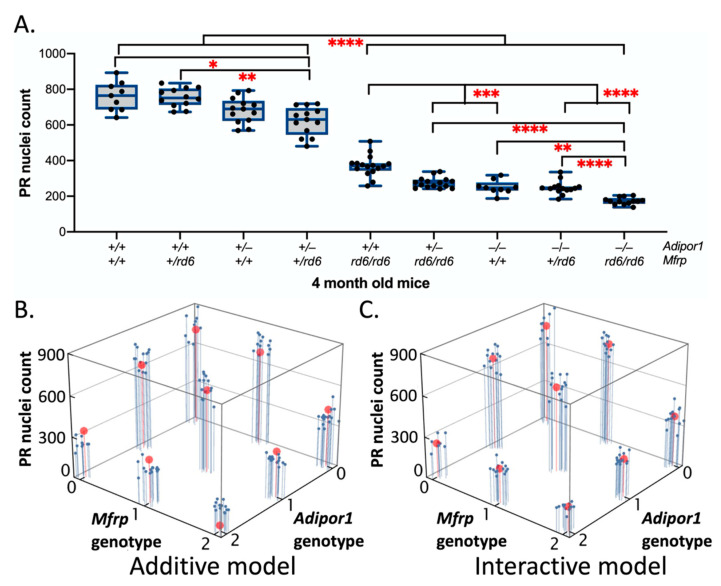
Photoreceptor degeneration in 4-month-old F2 mice from an (*Adipor1*^+*/*−^/*Mfrp^+/rd6^*) F1 intercross: (**A**) Using two-way factorial ANOVA, followed by Tukey’s HSD post-hoc analysis revealed statistically significant differences in PR degeneration between different genotypic possibilities of F2 intercross progeny. Absence of additive effect in single (*Adipor1^−/−^*/*Mfrp^+/+^*, *Adipor1*^+/+^/*Mfrp^rd6/rd6^*) and double (*Adipor1^−/−^*/*Mfrp^rd6/rd6^*) gene homozygous mutants, as compared to the wild types (*Adipor1^+/+^*/*Mfrp*^+/+^), confirmed the gene interaction effects. As no sex-based differences were found, data from both sexes were combined. * *p* < 0.05; ** *p* < 0.01; *** *p* < 0.001, and **** *p* < 0.0001. (**B**) Linear fit model without interactions and (**C**) linear fit model with interactions, showing actual data in blue and model predictions in red. The *Adipor1* and *Mfrp* genotypes along the two axis represent independent variables, where the alleles are scored as: wildtype—0, heterozygous—1, and homozygous—2. The PR nuclei count along the third axis represents response variables. The model predictions fit the actual data better in the interactive model, so it is concluded that the interactive model has a better fit than the additive model, confirming an interactive effect. *n* = 9–15 per genotype.

To examine the effect of individual genes as well as their interaction on PR degeneration, a linear model was again constructed using a full factorial design of the experiment with a standard least squares model, similar to the approach used for fundus spot and AL analysis. For PR nuclear count, hematoxylin and eosin-stained sections were used for measurements. Starting at the optic nerve (ON), the sections were divided into 300-micron-long intervals along the length of the retina, on both sides of the ON. Excluding the first 300-micron interval, the nuclei within the next 300-micron region of interest (ROI) were counted, and values were reported using a semi-automated approach as described in materials and methods. Final nuclei count values were reported as average nuclei counts within a 300-micron length on either side of the ON. For PR nuclear counts, an adjusted R^2^ value of 0.9384, RMSE of 0.1878, and a significant F Ratio of 216.4440 (prob > F = <0.0001) resulted from the interaction model revealing a significant main effect of both genes as well as a significant gene interaction effect on PR degeneration as shown in the effect tests table obtained from the JMP analysis (Supplementary Table S5). Tukey’s HSD post-hoc test was used to confirm the statistically significant differences in the PR degeneration between the nine genotypic possibilities for the *Adipor1/Mfrp* mutants as shown in Figure 4A. The parameter estimates obtained using statistical analysis (Supplementary Table S6) show significant effect of the zygosity in case of *Adipor1*, which suggests a semi-dominant nature of inheritance. The results were validated from the linear modeling using the R function, where the interaction and additive models were compared, and confirmed that the gene interaction model was a better fit than the purely additive model as shown in Figure 4B,C (adjusted R^2^ value for additive vs. interacting models: 0.846 vs. 0.938). An F-ratio test of the F-statistic derived from the two statistical models was significant, indicating superior performance of the interacting model (F-statistic: 43.7 and *p* = 7.5 × 10^–22^).

## 3. Discussion

Although both *Adipor1^tm1Dgen^* and *Mfrp^rd6^* mutant mice show very similar retinal disease characteristics, there is an incomplete understanding about the functional relationship between these two genes. Particularly, the individual contributions of *Adipor1* and *Mfrp* and their functional association to regulate phenotypes including fundus spots, AL changes, and PR degeneration have not been examined. Studies involving epistatic interactions, where the phenotypic impact of one gene depends on another gene, have been routinely used to expose functional associations [30,31]. We designed our study on the same principle, where the information obtained from epistatic interaction(s), inferred from the phenotypic measurements, was used to elucidate the potential functional association between *Adipor1^tm1Dgen^* and *Mfrp^rd6^*. We performed genetic interaction studies by comparing quantitative phenotypes of double mutant *Adipor1^tm1Dgen^ /Mfrp^rd6^* allelic combinations to single mutant (*Adipor1^tm1Dgen^* and *Mfrp^rd6^*) alleles, respectively. We measured three retinal phenotypes known to be affected in both *Adipor1^tm1Dgen^* and *Mfrp^rd6^* single gene mutants.

Both *Adipor1^tm1Dgen^* and *Mfrp^rd6^* homozygous mutants present with very similar spots by fundus photography. The white spots, for both mutants, correspond to the sub-retinally accumulated macrophages/monocytes, identified by immunolabelling with macrophage-specific markers MOMA-2 and F4/80+ [12,16,17,32]. This suggested the possibility that the genes may be functionally associated and follow similar biological processes of initiating an immune response, resulting in fundus spots. Our results from the epigenetic analysis confirmed significant individual gene as well as gene interaction effects on the spots phenotype. This shows that in addition to independent effects, perhaps through mutant-specific induced pathways, the fundus spotting due to loss of ADIPOR1 is also dependent on the fundus spotting due to loss of MFRP and vice versa, suggesting that some mechanistic aspects of the observed phenotype are also shared.

Similar to fundus spots, *Adipor1^tm1Dgen^* and *Mfrp^rd6^* homozygotes also show a significant decrease in PR cell count due to degeneration. Docosahexaenoic acid (DHA) is an important component of PR outer segment membranes and is vital for RPE and PR cell functions [33,34,35]. Studies have suggested that *Adipor1* acts as a regulatory switch for the uptake, retention, conservation, and elongation of DHA in PR and RPE cells, thus preserving PR cell integrity [17]. Recently, it has been suggested that *Mfrp* participates in DHA enrichment similar to *Adipor1* [24]. This suggests that these genes may be involved in regulating retinal lipidome homeostasis and that a decrease in DHA, very long chain-polyunsaturated fatty acids (VLC-PUFAs) and the inability to synthesize neuroprotective elovanoids along with the activation of inflammatory signaling pathways may lead to PR instability and degeneration [24,36] in both *Adipor1^tm1Dgen^* and *Mfrp^rd6^* homozygotes. Our results from the epigenetic analysis also confirmed significant individual gene as well as gene interaction effects on the PR degeneration. Our results demonstrate that both *Adipor1* and *Mfrp* are functionally associated for maintaining PR integrity, possibly through shared or different mechanisms that affect DHA enrichment.

For the *Adipor1* gene mutant, a significant effect of zygosity was observed for both fundus spots and PR degeneration phenotypes. While fundus spots are an expected phenotype in homozygous *Adipor1* and *Mfrp* mutants, it was interesting that the double heterozygous mutants as well as mice heterozygous for the *Adipor1* mutation (*Adipor1^+/−^/Mfrp^+/+^*) alone also developed a spotting phenotype. Similarly, a decrease in PR cell count was also observed in heterozygous *Adipor1* mice compared to age-matched controls. This confirmed that the presence of one wild type allele of *Adipor1* is not sufficient to prevent either of these phenotypes. This is in contrast with a previous study where no statistically significant differences were found in heterozygous and wild type *Adipor1* mice for the visual system protein levels measured, indicating that 50% of ADIPOR1 levels was sufficient for retinal stability [37]. However, that study was performed at P15 and P22. It is possible that the effect of zygosity is more pronounced with aging as is the case of our 4-month-old animals. Additionally, the observation of more fundus spots, at an earlier age in double heterozygous mice than in heterozygous *Adipor1* mice, suggests a possible interactive effect of *Mfrp* on the *Adipor1* phenotype. Concomitant with the increase in fundus spots is a greater relative decrease in PR nuclei in mutants bearing *Adipor1* loss-of-function alleles as compared to the mutants bearing loss of *Mfrp* alleles (Figure 2 and Figure 4). It is reasoned that, since homozygous *Mfrp^rd6^* mice lack ADIPOR1 protein in their RPE layer [37], it is likely that the partial RPE ADIPOR1 deficiency contributes to the PR degeneration observed in *Mfrp^rd6^* homozygotes. However, since ADIPOR1 is retained in the retina of *Mfrp^rd6^* homozygotes, there is relatively less PR degeneration at 4-months in *Mfrp^rd6^* homozygotes as compared to the *Adipor1^tm1Dgen^* homozygotes which have a complete loss of ADIPOR1 in both retinas as well RPE.

Mutations in *MFRP* have been associated with reduced AL [4,5,38]. A similar decrease in AL has also been reported in Zebrafish *Mfrp* mutants [39]. While initial studies with *rd6* and *rdx* mouse models did not report ocular AL abnormalities [13,16], later studies with *Mfrp* KI/KI mice [13] and *rd6* mice [4] demonstrated decreased ocular AL. Since *MFRP* contains a cysteine-rich domain essential for Wnt binding and signaling, which has been implicated in vertebrate eye development [40], studies have suggested the possibility that *MFRP* might regulate AL changes through Wnt signaling [2,13,41]. AL changes associated with *Adipor1* mutations have not been reported prior to this study. Our study confirms that, similar to *Mfrp^rd6^* mutants, *Adipor1^tm1Dgen^* mutants also exhibit decreased AL. However, in our epistatic analysis of AL in both the single and double mutants from the F2 cross, we did not observe gene interaction effects. Therefore, it appears the two genes affect AL in an independent, mutant-specific manner.

In summary, we illustrate that disruption in either *Adipor1* or *Mfrp* contributes significantly to the development of all three ocular phenotypes studied. Further, gene interaction modeling suggests that both mutant alleles interact to influence the severity of fundus spotting and PR cell degeneration, with no interaction effects on AL. Although, both *Adipor1^tm1Dgen^* and *Mfrp^rd6^* mutants share similar eye disease characteristics, the extent to which the phenotypes are affected differs in each of the mutants. This study highlights the complexity of functional associations and interactions between these two disease-associated genes, where they interact to exacerbate some phenotypes while contributing individually to others, and the analytical importance of epistatic studies in unravelling such associations.

## 4. Materials and Methods

### 4.1. Ethics Statement

Care and handling of mice in this study conformed to the Association for Research in Vision and Ophthalmology Resolution on the Use of Animals in Ophthalmic and Vision Research. All protocols involving mice were approved by The Jackson Laboratory Institutional Animal Care and Use Committee (IACUC, AUS99089), in accordance with the “Guide for the Care and Use of Experimental Animals” established by the National Institutes of Health (Bethesda, MD, USA).

### 4.2. Animals

Mouse strains used in this study, B6.129P2-*Adipor1^tm1Dgen^*/Mmnc (MMRRC, stock # 011599-UNC), B6.C3Ga- *Mfrp^rd6^*/J (The Jackson Laboratory, stock # 003684), and C57BL/6J (The Jackson Laboratory, stock # 000664), herein referred to as *Adipor1^−/−^*, *Mfrp^rd6/rd6^*, and wild type (WT), respectively, when used as double mutants, were bred and maintained under standard conditions of 12:12 light-dark cycle in the Research Animal Facility at The Jackson Laboratory. Mice were provided with NIH31 (6% fat chow) diet and HCl-acidified water (pH 2.8–3.2) ad libitum and maintained in pressurized individual ventilation caging, which was regularly monitored to ensure a pathogen-free environment. Both *Adipor1^−/−^* and *Mfrp^rd6^* are maintained on the C57BL/6J strain background and confirmed to be free of the *Crb1^rd8^* mutation.

To identify individual gene contributions and to ascertain the possibility of a digenic epistatic interaction between *Adipor1* and *Mfrp* gene mutations to modulate disease phenotypes, *Adipor1^tm1Dgen^* and *Mfrp^rd6^* homozygotes were bred together to generate F1 mice that were double heterozygotes (*Adipor1^+/−^*/*Mfrp^+/rd6^*). The resulting F1 offspring were then intercrossed to obtain an F2 generation to generate nine possible genotypic combinations (Supplementary Figure S3). All F2 mice were analyzed for the development of fundus spots, AL changes, and PR degeneration at 4-months of age.

### 4.3. Genotyping

Genomic DNA was isolated from either tail tips (<2 mm) or ear punches. DNA was extracted by incubating the tissue in 50 mM sodium hydroxide solution at 95 °C for 30–50 min, followed by the addition of 1M Tris HCl neutralizing reagent buffer to a final concentration of 200mM. The sample was briefly vortexed and spun at 3600 rpm for 8 min at 4 °C. Two µL of the supernatant containing the genomic DNA was then used per twelve µL PCR reaction reagents. For *Adipor1*, primers used were: NIH62-GS1, TCCACTGTGTCAGCTTCTCTGTTAC, NIH62-GS2, AGGCAGGGTAAGCTGATTAGCTATG, and NIH62-neo, GGGTGGGATTAGATAAATGCCTGCTCT (protocol from MMRRC at UNC #11599; www.med.unc.edu/mmrrc; accessed on 20 August 2021). Amplicons (254-bp for wild type and 433-bp for mutant) were visualized with EZ-vision In-Gel Solution (VWR, Catalog N391-15MLDRP) after electrophoretic separation on a 1.5% agarose gel. The *Mfrp^rd6^* mutation assay was carried out by the JAX genotyping facility using the available protocols (https://www.jax.org/Protocol?stockNumber=003684&protocolID=32162; accessed on 30 August 2021 and https://www.jax.org/Protocol?stockNumber=003684&protocolID=32010; accessed 30 August 2021).

### 4.4. Fundus Imaging

Fundus examination was performed as previously described [42], using a Micron IV fundus camera (Phoenix Research Laboratories, Pleasanton, CA, USA), with the exception that 1% cyclopentolate or 1% atropine was used as the dilating agent, and mice were anesthetized with isoflurane (isoflurane vaporizer from Kent Scientific, Torrington, CT, USA) for the duration of imaging.

### 4.5. Histological Analysis

Mice were euthanized by carbon dioxide asphyxiation. The enucleated eyes were placed in ice-cold methanol:acetic acid:PBS (3:1:4) solution for overnight fixation at 4 °C. The fixed eyes were subsequently paraffin embedded, cut into 4 µm sections, stained with hematoxylin and eosin, and visualized by light microscopy. Histological images were captured using a Nanozoomer digital slide scanner (Hamamatsu, Japan). The Fiji imaging processing package was used to assess PR cell loss from scanned images [43]. A custom Fiji macro was first used to extract .tif images of single sections from full-resolution NanoZoomer output files, which exceeded Fiji memory capabilities. A separate macro was then created to draw rectangular regions of interest (ROIs) encompassing a 300 µm length of retina and spaced at 0.3 mm intervals starting from the optic nerve head on either side. PR cell loss was determined from .tif images by counting PR cell nuclei within each ROI by both, using a macro as well as manually.

### 4.6. Axial Length Measurement

Enucleated eyes were assessed for axial length (anterior to posterior) using Vernier calipers. Three measurements were taken for each eye. Since the measurements were not found to differ statistically between right and left eyes and no sex-based differences were observed within each genotype (assessed by *t*-test), the measurements from both sexes were combined into one cohort per genotype to compare axial length measurements between different genotypes.

### 4.7. Refractive Error Measurement

The refractive state was measured in a darkened room with a custom-built automated eccentric infrared photo refractor calibrated according to a published procedure [44]. The data was recorded using software designed by Schaeffel et al. [44], and each measurement was repeated a minimum of three times. No statistically significant difference was observed between the right and left eyes (statistical significance determined by paired two-tailed Student’s *t*-test), thus, the data from both eyes of each animal was averaged.

### 4.8. Statistical Analysis

*t*-test and ANOVA were performed on data collected for fundus spotting (Supplementary Table S7), AL (Supplementary Table S8), and PR degeneration (Supplementary Table S9), using GraphPad Prism version 8 (GraphPad Software, San Deigo, CA, USA) and JMP statistical analysis software (SAS Institute, Cary, NC, USA). Individual gene contributions and genetic interaction effects were analyzed by two-way (factorial) ANOVA. Genes were selected as two categorical variables (factors) and the effect of their interaction was tested on each phenotype, selected as continuous (response) variable. To determine which means were different, a post-hoc Tukey’s HSD (for equal variances) or Dunnett’s T3 (for unequal variances) multiple comparison test was applied. The data were tested for normal distribution and equal variances and corrected for unequal sample sizes. Linear fit modeling was performed using R statistical software, version 3.4.3.

## Data Availability

The data presented in this study are available in the Supplementary Material.

## Supplementary Materials

The following supporting information can be downloaded at: https://www.mdpi.com/article/10.3390/ijms23031615/s1.
