# Supplementary material for: Genetic Interaction between Mfrp and Adipor1 Mutations Affect Retinal Disease Phenotypes
Source: Int J Mol Sci. 2022 Jan 30;23(3):1615. doi: 10.3390/ijms23031615 (PMC8835889; doi:10.3390/ijms23031615)
Supplement: Supplementary file 1 [file ijms-23-01615-s001.zip › ijms-1528552-supplementary.pdf]

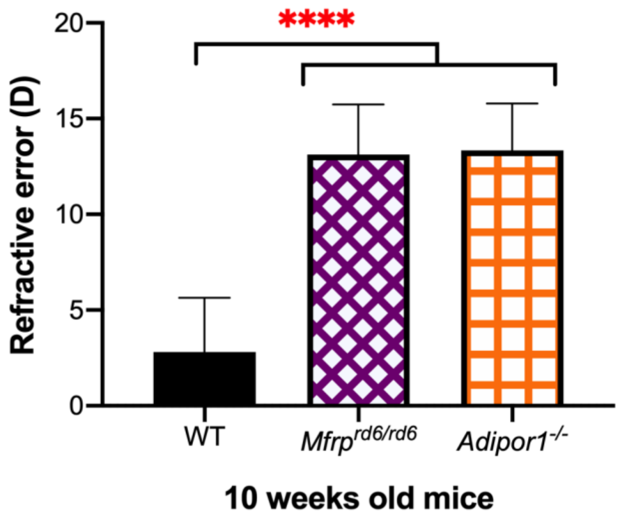

Figure S1: Significant changes in refractive error (Diopter) observed in *Adipor1*$^{-/-}$ and *Mfrp*^{rd6} mice, by 10 weeks of age, **** p > 0.0001.

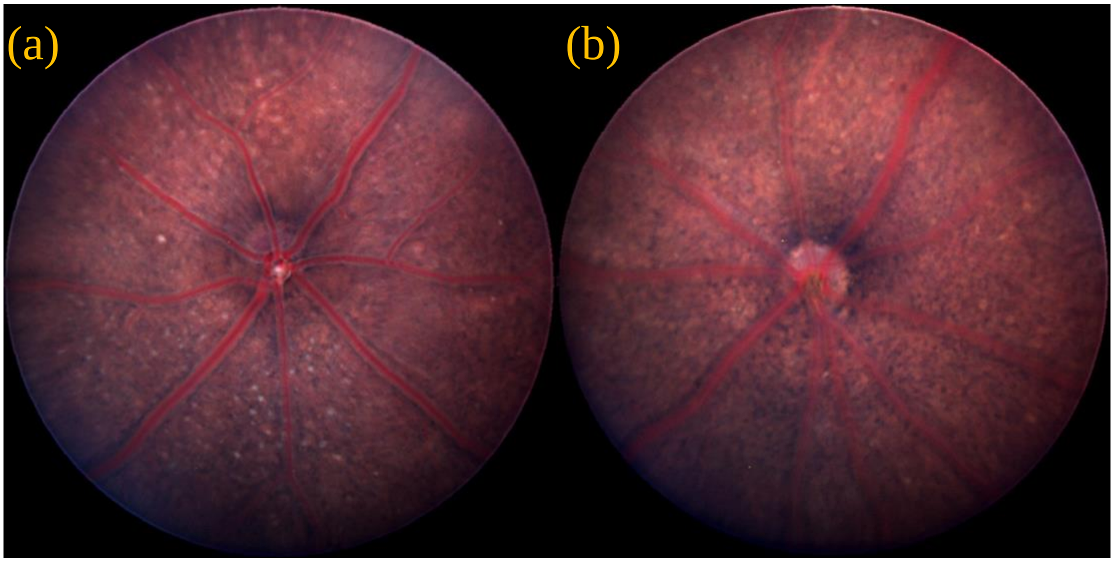

Figure S2: Spots observed by fundus photodocumentation in 10-week-old (a) double heterozygous (*Adipor1*$^{+/-}$/*Mfrp*$^{+/rd6}$) and (b) single heterozygous *Adipor1*$^{+/-}$ mice. N= 5.

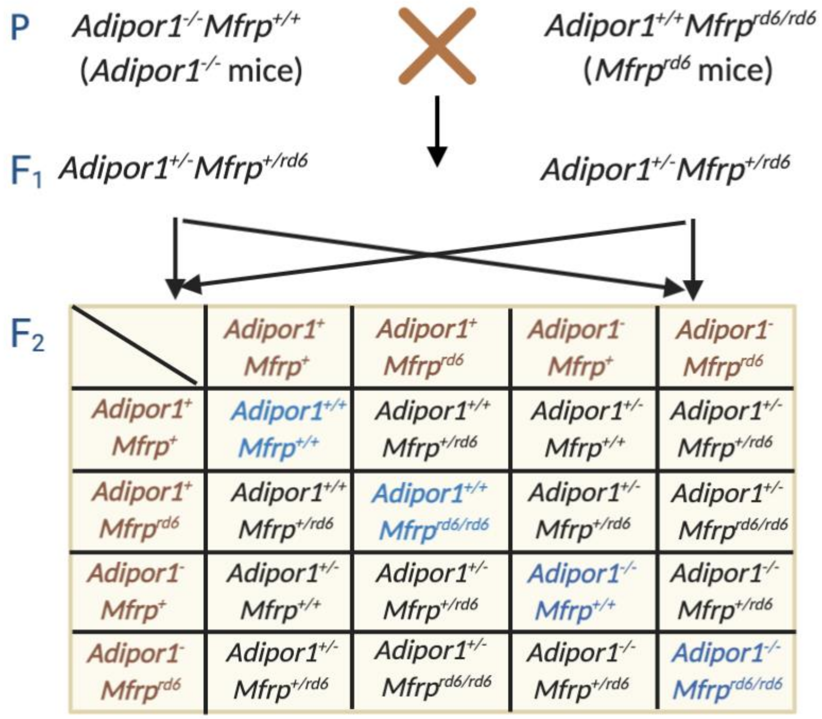

Figure S3: Generation of (*Adipor1*^{tm1Dgen} X *Mfrp*^{rd6}) F2 intercross progeny. Single gene *Adipor1*$^{-/-}$ and *Mfrp*^{rd6} mutant mice were crossed to produce *Adipor1/Mfrp* double heterozygous mice (F1). The double heterozygous mice were intercrossed to produce F2 mice with nine possible genotypic combinations as shown in the 16 Punnett square.

**Source**	**Nparm**	**DF**	**Sum of Squares**	**F Ratio**	**Prob>F**
Adipor1	2	2	1.7333333	18.5589	**<.0001**
Mfrp	2	2	2.6027778	27.8681	**<.0001**
Adipor1*Mfrp	4	4	1.8930577	10.1346	**<.0001**

Table S1: Effect tests obtained from fit model analysis of fundal spots, with the Prob>F = <0.0001 for both individual gene and gene interaction effects, confirming significant effect due to individual mutations as well as due to gene interaction.

Term	Estimate	Std Error	T Ratio	Prob>|t|
Intercept	0.2	0.096642	2.07	**0.0434**
Adipor1[1-0]	0.6	0.136672	4.39	**<.0001**
Adipor1[2-1]	0.2	0.136672	1.46	0.1493
Mfrp[1-0]	-0.075	0.123195	-0.61	0.5453
Mfrp[2-1]	0.875	0.123195	7.10	**<.0001**
Adipor1[1-0]*Mfrp[1-0]	0.275	0.17084	1.61	0.1134
Adipor1[1-0]*Mfrp[2-0]	-0.875	0.166222	-5.26	**<.0001**
Adipor1[2-1]*Mfrp[1-0]	-0.2	0.166222	-1.18	0.2417
Adipor1[2-1]*Mfrp[2-1]	-1.37e-15	0.151097	-0.00	1.0000

Table S2: Parameter estimates (the change in the response associated with a one-unit change of the predictor, all other predictors being held constant) obtained from fit test analysis for fundal spots, showing which variables/predictor have significant influence on the response. The genotypes were considered ordinal variables with 0 – Wild type; 1- heterozygous and 2- homozygous.

**Source**	**Nparm**	**DF**	**Sum of Squares**	**F Ratio**	**Prob>F**
Adipor1	2	2	15.308814	216.9900	**0.0074**
Mfrp	2	2	14.799119	209.7655	**<.0001**
Adipor1*Mfrp	4	4	5.946472	42.1432	0.5715

Table S3: Effect tests obtained from fit model analysis for axial length, with the Prob>F = <0.05 for both the individual gene contributions. Gene interaction effect is non-significant, confirming the absence of interaction between *Adipor1* and *Mfrp* for determining axial length.

Term	Estimate	Std Error	T Ratio	Prob>|t|
Intercept	3.2392857	0.012345	262.40	**<.0001**
Adipor1[1-0]	-0.00458	0.01667	-0.27	0.7839
Adipor1[2-1]	-0.055956	0.019804	-2.83	**0.0053**
Mfrp[1-0]	-0.005649	0.015791	-0.36	0.7210
Mfrp[2-1]	-0.107922	0.014092	-7.66	**<.0001**
Adipor1[1-0]*Mfrp[1-0]	-0.000857	0.020434	-0.04	0.9666
Adipor1[1-0]*Mfrp[2-0]	0.0177221	0.018653	0.95	0.3434
Adipor1[2-1]*Mfrp[1-0]	0.0110892	0.024023	0.46	0.6449
Adipor1[2-1]*Mfrp[2-1]	0.0111524	0.021071	0.53	0.5973

Table S4: Parameter estimates (the change in the response associated with a one-unit change of the predictor, all other predictors being held constant) obtained from fit test analysis for axial length, showing which variables/predictor have significant influence on the response. The genotypes were considered ordinal variables with 0 – Wild type; 1- heterozygous and 2- homozygous.

**Source**	**Nparm**	**DF**	**Sum of Squares**	**F Ratio**	**Prob>F**
Adipor1	2	2	15.308814	216.9900	**<.0001**
Mfrp	2	2	14.799119	209.7655	**<.0001**
*Adipor1*Mfrp*	4	4	5.946472	42.1432	**<.0001**

Table S5: Effect Tests obtained from fit model analysis for PR degeneration, with the Prob>F = <0.0001 for both individual gene and gene interaction effects, confirming significant effect due to individual mutations as well as due to gene interaction.

Term	Estimate	Std Error	T Ratio	Prob>|t|
Intercept	2.5097667	0.062606	40.09	**<.0001**
Adipor1[1-0]	-0.239121	0.081443	-2.94	**0.0041**
Adipor1[2-1]	-1.434896	0.081443	-17.62	**<.0001**
Mfrp[1-0]	0.0041458	0.08282	0.05	0.9602
Mfrp[2-1]	-1.278356	0.071724	-17.82	**<.0001**
Adipor1[1-0]*Mfrp[1-0]	-0.20185	0.110843	-1.82	0.0714
Adipor1[1-0]*Mfrp[2-0]	0.1241006	0.101042	1.23	0.2221
Adipor1[2-1]*Mfrp[1-0]	0.1884038	0.108932	1.73	0.0866
Adipor1[2-1]*Mfrp[2-1]	0.9040249	0.101481	8.91	**<.0001**

Table S6: Parameter Estimates (the change in the response associated with a one-unit change of the predictor, all other predictors being held constant) obtained from fit test analysis for PR degeneration, showing which variables/predictor have significant influence on the response. The near-zero coefficients indicate the variables with very little influence on the response. The variables with Prob>|t|= <0.05 are considered to have significant influence on response (PR degeneration in this case). The genotypes were considered ordinal variables with 0 – Wild type; 1-heterozygous and 2- homozygous.

Adipor1	Mfrp	Spots
0	0	0
0	0	0
0	0	0
0	0	1
0	0	0
0	1	0
0	1	0
0	1	1
0	1	0
0	1	0
0	1	0
0	1	0
0	1	0
1	0	1
1	0	0
1	0	1
1	0	1
1	0	1
1	1	1
1	1	1
1	1	1

1	1	1
1	1	1
1	1	1
1	1	1
1	1	1
1	1	1
1	1	1
0	2	1
0	2	1
0	2	1
0	2	1
0	2	1
1	2	1
1	2	1
1	2	1
1	2	1
1	2	1
1	2	1
2	0	1
2	0	1
2	0	1
2	0	1
2	0	1
2	1	1
2	1	1
2	1	1
2	1	1
2	1	1
2	1	1
2	1	1
2	1	1
2	1	1
2	2	1
2	2	1
2	2	1
2	2	1
2	2	1
2	2	1
2	2	1

2	2	1
2	2	1

Supplementary Table S7: Data from fundus spot analysis: Nine genotypic combinations of *Adipor1/Mfrp* double mutants used as independent variables, and data from fundus spot used as a response variable for factorial ANOVA. The genotypes were given variables with 0 – Wild type; 1- heterozygous and 2- homozygous.

Adipor1	Mfrp	Axial length
0	0	3.27
0	0	3.27
0	0	3.18
0	0	3.26
0	0	3.25
0	0	3.27
0	0	3.24
0	0	3.2
0	0	3.2
0	0	3.25
0	0	3.27
0	0	3.23
0	0	3.23
0	0	3.23
0	1	3.27
0	1	3.22
0	1	3.14
0	1	3.15
0	1	3.2
0	1	3.27
0	1	3.25
0	1	3.26
0	1	3.23
0	1	3.29
0	1	3.32
0	1	3.28
0	1	3.26
0	1	3.24
0	1	3.26

0	1	3.29
0	1	3.24
0	1	3.2
0	1	3.17
0	1	3.13
0	1	3.25
0	1	3.22
1	0	3.15
1	0	3.27
1	0	3.29
1	0	3.27
1	0	3.23
1	0	3.26
1	0	3.24
1	0	3.3
1	0	3.29
1	0	3.28
1	0	3.21
1	0	3.19
1	0	3.29
1	0	3.14
1	0	3.22
1	0	3.17
1	0	3.19
1	1	3.23
1	1	3.24
1	1	3.25
1	1	3.2
1	1	3.3
1	1	3.19
1	1	3.25
1	1	3.15
1	1	3.27
1	1	3.15
1	1	3.14
1	1	3.24
1	1	3.21
1	1	3.13
1	1	3.26

1	1	3.25
1	1	3.24
1	1	3.2
1	1	3.27
1	1	3.25
1	1	3.22
1	1	3.32
1	1	3.25
1	1	3.32
1	1	3.26
1	1	3.2
1	1	3.26
1	1	3.28
1	1	3.2
1	1	3.26
1	1	3.24
1	1	3.14
1	1	3.25
1	1	3.24
1	1	3.3
1	1	3.23
1	1	3.27
1	1	3.28
1	1	3.17
1	1	3.29
1	1	3.24
1	1	3.15
1	1	3.23
1	1	3.14
1	1	3.16
1	1	3.22
1	1	3.29
1	1	3.24
1	1	3.19
1	1	3.15
0	2	3.1
0	2	3.1
0	2	3.11
0	2	3.15

0	2	3.07
0	2	3.07
0	2	3.08
0	2	3.05
0	2	3.19
0	2	3.18
0	2	3.16
0	2	3.17
0	2	3.15
0	2	3.18
0	2	3.08
0	2	3.14
0	2	3.13
0	2	3.16
0	2	3.14
0	2	3.13
0	2	3.1
1	2	3.18
1	2	3.13
1	2	3.12
1	2	3.14
1	2	3.1
1	2	3.12
1	2	3.17
1	2	3.11
1	2	3.18
1	2	3.17
1	2	3.19
1	2	3.14
1	2	3.19
1	2	3.06
1	2	3.1
1	2	3.18
1	2	3.09
1	2	3.13
1	2	3.14
1	2	3.12
2	0	3.14
2	0	3.11

2	0	3.17
2	0	3.19
2	0	3.24
2	0	3.13
2	0	3.2
2	0	3.25
2	1	3.14
2	1	3.18
2	1	3.19
2	1	3.22
2	1	3.21
2	1	3.2
2	1	3.18
2	1	3.14
2	1	3.24
2	1	3.11
2	1	3.16
2	1	3.19
2	1	3.23
2	1	3.22
2	1	3.14
2	2	3.06
2	2	3.16
2	2	3.07
2	2	3.04
2	2	3.02
2	2	3.09
2	2	3.11
2	2	3.07
2	2	3.12
2	2	3.11
2	2	3.18
2	2	3.19
2	2	3.11
2	2	3.13

Supplementary Table S8: Data for axial length measurement: Nine genotypic combinations of *Adipor1/Mfrp* double mutants used as independent variables, and axial length values used as response variable for factorial ANOVA. The genotypes were given variables with 0 – Wild type; 1- heterozygous and 2- homozygous.

Adipor1	Mfrp	Degeneration
0	0	2.42275
0	0	2.0892
0	0	2.13795
0	0	2.5481
0	0	2.9754
0	0	2.28405
0	0	2.72015
0	0	2.6268
0	0	2.7835
1	0	2.0561
1	0	2.22615
1	0	1.8972
1	0	1.91605
1	0	2.37375
1	0	2.08895
1	0	2.64365
1	0	2.20335
1	0	2.49975
1	0	2.40045
1	0	2.60085
1	0	2.31475
1	0	2.2974
0	1	2.69445
0	1	2.609
0	1	2.6468
0	1	2.405
0	1	2.4836
0	1	2.78175
0	1	2.5266
0	1	2.7052
0	1	2.3637
0	1	2.4565
0	1	2.2522
0	1	2.24215
1	1	1.7352
1	1	2.04475
1	1	1.602
1	1	2.1799

1	1	2.25375
1	1	2.10175
1	1	2.39535
1	1	2.3806
1	1	2.3753
1	1	1.8931
1	1	2.21065
1	1	1.73435
1	1	2.04155
2	1	1.0181
2	1	0.8398
2	1	0.6133
2	1	0.8096
2	1	0.8088
2	1	1.11765
2	1	0.8202
2	1	0.79155
2	1	0.842
2	1	0.8644
2	1	0.6809
2	1	0.8162
2	1	0.78275
2	1	0.76505
2	0	0.7912
2	0	0.8514
2	0	0.6231
2	0	0.83225
2	0	0.7795
2	0	1.06085
2	0	0.7645
2	0	0.99525
2	0	0.8237
0	2	1.69335
0	2	0.92985
0	2	1.2851
0	2	1.2436
0	2	1.5099
0	2	1.22505
0	2	1.2739

0	2	1.4026
0	2	1.2816
0	2	1.25285
0	2	1.21845
0	2	1.1887
0	2	1.1814
0	2	0.86005
0	2	1.09275
0	2	1.12975
1	2	0.9421
1	2	0.98605
1	2	0.83615
1	2	1.12595
1	2	1.1025
1	2	0.8048
1	2	0.9176
1	2	0.8773
1	2	0.9759
1	2	0.8569
1	2	0.9467
1	2	0.85795
1	2	0.92605
1	2	0.8136
1	2	0.81075
2	2	0.5882
2	2	0.5703
2	2	0.5695
2	2	0.5357
2	2	0.6014
2	2	0.66885
2	2	0.571
2	2	0.5088
2	2	0.5111
2	2	0.68265
2	2	0.4601
2	2	0.5615
2	2	0.66175

Supplementary Table S9: Data for PR degeneration: Nine genotypic combinations of *Adipor1/Mfrp* double mutants used as independent variables, and PR nuclear count values used as response variable for factorial ANOVA. The genotypes were given variables with 0 – Wild type; 1- heterozygous and 2- homozygous.
